# Classroom-Based Physical Activity Breaks and Children's Attention: Cognitive Engagement Works!

**DOI:** 10.3389/fpsyg.2016.01474

**Published:** 2016-10-04

**Authors:** Mirko Schmidt, Valentin Benzing, Mario Kamer

**Affiliations:** Institute of Sport Science, University of BernBern, Switzerland

**Keywords:** cognition, acute exercise, mediation, executive functions, affect, PANAS, mental effort, school

## Abstract

Classroom-based physical activity breaks are postulated to positively impact children's attention during their school day. However, empirical evidence for this claim is scarce and the role of cognitive engagement in enhancing children's attentional performance is unexplored in studies on physical activity breaks. The aim of the present study was therefore to disentangle the separate and/or combined effects of physical exertion and cognitive engagement induced by physical activity breaks on primary school children's attention. In addition, the role of children's affective reactions to acute interventions at school was investigated. Using a 2 × 2 between-subjects experimental design, 92 children between the ages of 11 and 12 years (*M* = 11.77, *SD* = 0.41) were randomly assigned to one of four experimental conditions: (1) combo group (physical activity with high cognitive demands), (2) cognition group (sedentary with high cognitive demands), (3) physical group (physical activity with low cognitive demands), and (4) control group (sedentary with low cognitive demands). Attention and affect were measured before and immediately after a 10-min intervention. ANCOVAs revealed that whereas physical exertion had no effect on any measure of children's attentional performance, cognitive engagement was the crucial factor leading to increased focused attention and enhanced processing speed. Mediational analyses showed that changes in positive affect during the interventions mediated the effect between cognitive engagement and focused attention as well as between cognitive engagement and processing speed. These surprising results are discussed in the light of theories predicting both facilitating and deteriorative effects of positive affect on attention.

## Introduction

“Everyone knows what attention is. It is the taking possession by the mind, in clear and vivid form, of one out of what seem several simultaneously possible objects or trains of thought. Focalization, concentration, of consciousness are of its essence” (James, 1890, pp. 403–404).

Inspired by the seminal work of William James (1890), many scientists have studied the construct of attention, resulting in almost as many different theoretical approaches and definitions (e.g., Treisman and Gelade, 1980; Posner and Petersen, 1990; Corbetta and Shulman, 2002). Most researchers, however, claim a multi-component nature of attention and distinguish between attentional orienting, divided, sustained and selective attention (Coull, 1998). The expressions *inhibitory control of attention, executive attention, concentration* or *focused attention* are used interchangeably to describe attentional processes that are driven by a voluntary component, which tries to ignore certain stimuli while attending to others (Posner and DiGirolamo, 1998). Though not uniformly accepted, focused attention is mostly considered to be a part of inhibitory control, being one of the three core executive functions—alongside working memory and cognitive flexibility (Barkley, 1996; Posner and DiGirolamo, 1998; Diamond, 2013). Focused attention is an important prerequisite for learning (Steinmayr et al., 2010) and has high long-term predictive validity for children's academic achievement (Steele et al., 2012). Due to its relevance for the entire learning process in school, ecologically feasible interventions that promote children's attention are often called for (Stylianou et al., 2016).

Acute bouts of physical activity seem to be a promising way to immediately enhance children's attentional performance (Hillman et al., 2011; Chang et al., 2012; Verburgh et al., 2014). The interventions applied in the constantly growing body of studies on the effects of physical activity on children's attention vary not only in their quantitative characteristics, such as intensity and duration, but also in their qualitative characteristics, such as physical activity modality. Whereas investigations into intensity and duration have resulted in prescribing 11–20 min of exercise at moderate to vigorous intensity as having the most facilitating effect on various measures of cognitive performance (Chang et al., 2012, 2015), empirical evidence is limited and contradictory when it comes to its qualitative characteristics (Pesce and Ben-Soussan, 2016). One of the qualitative characteristics of physical activity most widely discussed as having an impact on children's attention is the cognitive demand inherent in many forms of physical activity (Best, 2010). This cognitive demand is thought to induce cognitive engagement (CE), defined as the degree to which the allocation of attentional resources and cognitive effort is needed to master difficult skills (Tomporowski et al., 2015). CE, in turn, is supposed to lead to better attention by pre-activating the same cognitive processes during physical activity as the ones used in a subsequent cognitive task (Budde et al., 2008). When, for example, playing hopscotch requires the ability to discriminate between different visual stimuli and to make appropriate motor decisions, performance should, as a result, be facilitated in a consecutive cognitive test involving exactly the same cognitive processes. Results from basic research seem to support this postulated mechanism, in which particularly complex motor tasks should be appropriate to investigate the “link between action and cognition” (Serrien et al., 2007). However, some studies that have manipulated CE of physical activity in children and adolescents have revealed positive effects on attentional performance in favor of the cognitively engaging condition (Budde et al., 2008; Pesce et al., 2009; Jäger et al., 2014; Benzing et al., in review), while others have found no difference concerning the overall study sample (Best, 2012; Jäger et al., 2015) or even detrimental effects compared to physical activity without CE (Gallotta et al., 2012, 2015).

A closer examination of those studies which varied the level of CE, controlling for exercise intensity and duration, divulges procedural differences that might be responsible for the inconsistent findings. The intensity level and duration vary widely across the studies, with heart rates ranging from 120 (Budde et al., 2008) to 160 bpm (Best, 2012) and activity durations ranging from 10 (Budde et al., 2008) to 50 min (Gallotta et al., 2012, 2015). Besides attention (Budde et al., 2008; Best, 2012; Gallotta et al., 2012, 2015; Jäger et al., 2015), working memory (Pesce et al., 2009; Jäger et al., 2015) and cognitive flexibility (Jäger et al., 2015; Benzing et al., in review) were regarded as primary outcomes of the studies. An accurate comparison of the reported studies is hampered by the fact that different dependent variables are reported in them. In terms of the modality, different kinds of cognitively engaging forms of physical activity (e.g., coordinative exercise, team games, exergaming) were examined using either one comparison group—a less cognitively engaging physical activity only (Budde et al., 2008; Pesce et al., 2009); two comparison groups—a less cognitively engaging physical activity and a passive control group (Gallotta et al., 2012, 2015; Benzing et al., in review); or three comparison groups—a less cognitively engaging physical activity, a cognitively engaging sedentary condition and a passive control group (Best, 2012; Jäger et al., 2015). Clearly, a study design using four experimental conditions is more appropriate to explore the role of cognitive engagement induced by acute physical activity, as it additionally considers potential effects of a sedentary cognitive condition. The only two studies using such a 2 × 2 design, however, produced mixed results, with Best (2012) revealing a main effect for the physical component and Jäger et al. (2015) finding neither a main effect for physical activity nor for CE. These mixed results prevent the question, whether physical exertion (PE), CE or both in combination are most beneficial for children's attention, from being conclusively answered.

In the school context, classroom-based physical activity aims to improve both physical activity level and academic achievement (Donnelly and Lambourne, 2011). It has been demonstrated that applying classroom-based physical activity programs enhances physical activity levels (e.g., Kibbe et al., 2011) and reduces sedentary time (Riley et al., 2015). Empirical evidence concerning the effects of classroom-based physical activity on variables related to academic achievement suggests that it is effective at enhancing children's enjoyment (Vazou and Smiley-Oyen, 2014), their cognitive function (Hill et al., 2010) and their on-task behavior (Mahar, 2011), as well as their attention (Palmer et al., 2013). Classroom-based physical activity itself can be further distinguished into (1) integrated physical activity, incorporating physical activity *during* academic lessons, e.g., having students walking on a balance beam while trying to solve a math problem, and (2) physical activity breaks, consisting of short bouts of physical activity *between* lessons, e.g., having students perform coordinative exercises between two consecutive academic lessons (Webster et al., 2015). To date, most of the research has focused on chronic, i.e., long-term interventions, rather than acute, i.e., single bouts of classroom-based physical activity, and on integrated physical activity rather than on physical activity breaks. Since the implementation of physical activity breaks is relatively simple and effects of single bouts of physical activity are highly relevant for academic achievement, it is surprising that only few studies have investigated the effects of acute physical activity breaks.

The literature on physical activity breaks, typically consisting of short bouts (5–15 min) of physical activity at moderate intensity levels, is contradictory and limited to very few studies. Whereas studies using shorter durations (5 min) have consistently been unable to detect improvements in inhibition (Kubesch et al., 2009) or in on-task behavior (Howie et al., 2014), most studies using longer activity breaks (10–15 min) reported positive effects on the assessed cognitive measures. Hill et al. (2010), Janssen et al. (2014) and Howie et al. (2014) reported beneficial effects on attention, on-task behavior and working memory. In contrast, Wilson et al. (2016) reported no effects on sustained attention and on-task behavior. Aside from the varying durations, one reason for the mixed results might also be the different types of physical activity breaks used. Although not explicitly stated, none of the interventions that found positive effects consisted of “pure” aerobic exercises, but instead involved cognitively challenging activities such as “passing of the ball and dribbling with the ball” (Janssen et al., 2014, p. 130) or “hopping sequences to music” (Hill et al., 2010, p. 930). In that sense, enhanced social interactions or CE through mastering challenging tasks could have affected children's attention in the experimental group. Thus, one could hypothesize that physical activity breaks that combine physical effort with cognitive demands would produce effects beyond those of pure aerobic exercise, e.g., running in place. The answer to the question which specific forms of physical activity breaks would be most beneficial for children's attention would be of great practical importance in the educational setting and in particular, for designing school-based physical activity programs that target children's attention.

Aside from CE, it has recently been argued that positive mood or affect could also mediate the relationship between acute physical activity and cognition (Audiffren and André, 2015). This conceivable, yet untested, hypothesis is based on the results of a continuously growing body of studies showing that positive affect is both a result of acute physical activity (Reed and Ones, 2006; Ekkekakis et al., 2011) and a predictor of attention (Forgas and Eich, 2012). Concerning the relationship between physical activity and positive affect, there seems to be consensus that sub-threshold intensities ranging from 10 to 30 min cause pleasant changes for most individuals, intensities close to the ventilatory threshold lead to large inter-individual variability and supra-threshold intensities result in uniformly negative changes in affective reactions (Ekkekakis et al., 2011). With respect to the relationship between positive affect and attention, the evidence is equivocal. The basic assumption made by Audiffren and André (2015) is that positive affect allows individuals to go beyond their usual limits in cognitive tasks, leading to enhanced attentional performance. However, looking for theories that explain the relationship between affect and cognition, it becomes apparent that the proposed facilitative effect of positive affect on attention is only partly supported, calling for a differentiated presentation of these theories.

The three theories discussed in the literature lead to different predictions concerning the relationship between affect and attention (Mitchell and Phillips, 2007). *Capacity limitation theories* assume that both positive and negative affective mood states—as compared with neutral states—adversely affect attention, because they require additional resources (Seibert and Ellis, 1991). It is therefore postulated that fewer resources will be available for cognitively demanding tasks (Mitchell and Phillips, 2007). The *mood as information theory* postulates that positive affect—as compared to neutral affect states—is associated with a more heuristic processing style. Heuristic processing involves a non-rigorous problem-solving approach governed by availability, accessibility, and applicability of information, using shortcuts to reach an answer. Therefore, this style is characterized by less precise but faster information processing, which should have a negative effect on tasks that require attention to be focused on specific elements of the task, such as in attention tests. Negative mood, on the other hand, signalizes a problematic situation and induces an analytical processing style. Analytic processing is characterized by a more careful, systematic and in-depth treatment of information, narrowing the available action tendencies. This focus on individual task elements should further lead to enhanced performance in attentional control tasks (Schwarz, 1990). Along similar lines, *mood as a facilitator theories* assume that positive affect activates a set of positive memories and thoughts, which in turn lead to a more flexible and innovative problem-solving style (Isen, 1999; Isen and Reeve, 2005). Studies based on this group of theories show that especially cognitive flexibility can be positively influenced by positive mood (Isen, 2008) and predict that especially in interesting and novel tasks, performance will be enhanced. In conclusion, all these theories suggest that affective mood states, as for example induced by physical activity, could influence attentional performance. Nonetheless, predictions of whether this influence is facilitative or deteriorative vary between theories, leaving the respective question unanswered.

The three aims of the present study were therefore to test whether (1) PE and CE impact children's attention separately or in combination; (2) positive affect has an effect on children's attention; and (3) a potential relationship between the two manipulated variables (PE and CE) and attentional performance was mediated by changes in positive affect.

## Materials and methods

### Overview

To test the aforementioned study hypotheses, the present study used a 2 × 2 between-subjects experimental design. Children were randomly assigned to one of four experimental conditions: (1) combo group (physical activity with high cognitive demands), (2) cognition group (sedentary with high cognitive demands), (3) physical group (physical activity with low cognitive demands), (4) control group (sedentary with low cognitive demands). Attention and affect were measured before (pre-test) and immediately after the intervention (post-test). To test whether the manipulation of physical exertion (PE) was successful, first, the children's heart rate was assessed throughout the entire intervention; second, ratings of perceived physical exertion (RPE) were determined after the intervention. To test the successful manipulation of cognitive engagement (CE), ratings of perceived cognitive engagement (RCE) were also collected after the intervention. Information on the following background variables was gathered after the post-test: age, gender, pubertal status, socioeconomic status, and physical activity level, as well as height and weight for calculating the body mass index (BMI).

### Participants

A total of 98 fifth grade primary school children from 5 different classes in the region of Bern (Switzerland) took part in the study and were randomly assigned to one of the four conditions. Four participants were excluded because their accuracy score on the d2-R test of attention (see Experimental Measures) was above the cut-off point of 20% recommended by Brickenkamp et al. (2010). Two children were identified as probable multivariate outliers based on the Mahalanobis distance (Fidell and Tabachnick, 2003) and were therefore excluded as well. Since the MCAR test according to Little was not significant [χ^2^ (580) = 576.87, *p* = 0.53], all missing values (4.92%) were imputed using the expectation-maximization algorithm. Thus, the final sample consisted of 92 children (45.7% girls) between 11.01 and 12.98 years (*M* = 11.77, *SD* = 0.41 years). The children's parents signed an informed consent form approved by the Institutional Review Board. Immediately before testing, the children were again asked whether they wanted to participate and they were informed that they could discontinue at any time during the study. All data was treated confidentially.

In view of previous studies that tested the effects of an acute physical activity intervention on the d2 Test of Attention with participants of a comparable age (Gallotta et al., 2015), an a priori power analysis was performed with power (1–beta error probability) = 0.80, alpha error probability = 0.05, effect size *f* = 0.38, numerator *df* = 5, number of groups = 4, and number of covariates = 1, resulting in an optimal sample size of 95.

There were no significant differences with respect to any of the background variables (age, gender distribution, pubertal status, socioeconomic status, physical activity level, BMI), the baseline levels of the dependent variables or the covariate between the participants of the four groups (Table 1).

**Table 1:** **Means (standard deviations in parenthesis) and test statistics for the background and manipulation check variables of the four conditions**.

	**High CE**		**Low CE**		***F*_(3, 87)_**	***p***	**$\eta_{\text{p}}^{2}$**
	**Combo group**	**Cognition group**	**Physical group**	**Control group**			
**SAMPLE CHARACTERISTICS**							
Age (years)	11.80 (0.42)	11.75 (0.34)	11.77 (0.43)	11.77 (0.41)	0.07	0.975	
Female %	48.0%	45.5%	44.0%	45.0%	0.03	0.994	
Pubertal status	5.09 (1.41)	5.36 (1.59)	5.00 (1.57)	5.37 (1.48)	0.35	0.789	
Socioeconomic status	7.24 (1.23)	6.86 (1.46)	7.01 (1.46)	6.41 (1.60)	1.32	0.270	
Physical activity level	145.69 (114.29)	180.92 (146.86)	201.09 (152.57)	268.86 (202.09)	2.44	0.069	
Body mass index	18.74 (2.86)	17.85 (3.09)	17.49 (2.70)	18.16 (2.52)	0.89	0.452	
**MANIPULATION CHECK VARIABLES**							
Heart rate (bpm)	154.05 (25.73)	102.92 (21.07)	144.63 (35.40)	87.93 (9.81)	35.41	<0.0005	0.55
Rating of perceived physical exertion (RPE)	12.04 (3.54)	9.14 (2.40)	11.08 (3.14)	6.80 (1.82)	15.17	<0.0005	0.33
Rating of perceived cognitive engagement (RCE)	11.08 (2.50)	11.09 (2.62)	8.84 (3.20)	8.65 (3.47)	4.86	0.004	0.14

### Procedure

The data was collected over a period of 3 weeks on five different mornings at precisely the same time. At the beginning of the testing (10.15 a.m.–10.25 a.m.), participants were informed about the procedure for the study and familiarized with the d2-R (Brickenkamp et al., 2010), the test used to assess focused attention. After 20 min of standardized German language teaching (10.25 a.m.–10.45 a.m.), the pre-test was performed (10.45 a.m.–10.55), which included the d2-R and the short version of the PANAS-C (Ebesutani et al., 2012). To make the study as ecologically valid as possible, the pre-test was followed by another 20 min of language class (10.55 a.m.–11.15 a.m.) before the four interventions, each lasting 10 min, took place (11.15 a.m.–11.30 a.m.; including 5 min for instructions and small reorganizations of the classroom). Immediately after the treatment (11.30 a.m.–11.40 a.m.), the post-test was carried out, again consisting of the d2-R and the PANAS-C, as well as a rating of perceived physical exertion (RPE) using the Borg RPE scale (Borg, 1998) and an adapted version for assessing perceived cognitive engagement (RCE). Each measure is discussed in detail below.

### Experimental conditions

#### Combo group (physical activity with high cognitive demands; *n* = 25, 48.0% girls, *M* = 11.80 years, *SD* = 0.42):

The physical activity for this condition was created on the basis of the “number connection test” (Zahlenverbindungstest, ZVT; Oswald and Roth, 1987), which was used in the treatment of the *cognition group*. The children had to touch numbers from 1 to 18, which had been randomly painted on the ground of an area measuring 5 by 5 meters. They were asked to touch the numbers in ascending order as quickly as possible. Once they had touched all the 18 numbers, they had to start again. After a period of 5 min, the children had to compute their total score, before having to try and improve during a second 5-min period. The number of repetitions varied between four and seven times, depending on the child. CE was thought to be induced by the demands of this physical version of the number connection test, requiring the subject to discriminate simple visual stimuli, perform fast mental operations and react with an appropriate motor response.

#### Cognition group (sedentary with high cognitive demands; *n* = 22, 45.5% girls, *M* = 11.75 years, *SD* = 0.34):

Children worked on the ZVT (Oswald and Roth, 1987), a paper-and-pencil trail-making test. Like the d2-R, the ZVT makes high demands on focused attention (Brickenkamp et al., 2010). The participants received several matrices with numbers from 1 to 90. They were asked to draw a line between the numbers in ascending order as quickly as possible. After a period of 5 min, the children had to compute their total score, before they had to try and improve during a second 5-min period.

#### Physical group (physical activity with low cognitive demands; *n* = 25, 44.0% girls; *M* = 11.77 years, *SD* = 0.43):

This condition consisted of 10 min of running at different speeds. While running around, children had to imagine that they were changing gear in a car, whereby they changed their running speed. The moment of the “gear change” was announced by the investigator. In contrast to the task of the *combo group*, the cognitive demands were minimized in this condition but the physical intensity was intended to be similar.

#### Control group (sedentary with low cognitive demands; *n* = 20, 45.0% girls, *M* = 11.77 years, *SD* = 0.41):

Children remained at their desks in the classroom and listened 10 min to an age-appropriate story. To keep the cognitive demands as low as possible, the children were told that they would not be tested on the contents of the story and that they could relax and enjoy.

### Manipulation check variables

Polar Team^2^ belts and transmitters (Polar Electro Oy, Kempele, Finland) were used to measure children's heart rate in all four experimental conditions. In the analyses, the mean heart rate during the intervention period was used as a measure of *physical exertion* (PE). In addition, the perceived physical exertion (RPE) was measured using the Borg RPE scale for perceived physical exertion (Borg, 1998). Evidence for the acceptable reliability and validity of the Borg RPE scale in preadolescents has been provided by Lamb (1996).

In order to determine the *cognitive engagement* (CE) of the children during the treatments, the Borg RPE scale was adapted to specifically ask for perceived cognitive engagement (RCE) of the activity. As in the Borg RPE scale, the participants had to rate their exertion ranging from 6 (“no exertion at all”) to 20 (“maximal exertion”). The question they had to answer was “How exhausting was the previous activity for your brain?” This adapted version is not a validated instrument, but proved to be feasible in a previous study in adolescents (Benzing et al., in review).

### Experimental measures

To assess *attention*, the d2-R test of attention (Brickenkamp et al., 2010), which is the revised version of the d2 Test of Attention (Brickenkamp and Zillmer, 1998), was used. The d2-R is a paper-and-pencil letter-cancelation test that consists of 14 lines of 57 randomly mixed “p”s and “d”s with one to four single quotation marks either above and/or below each letter. Within 20 s for each line, respondents are asked to strike out only the letter “d” with two dashes and to ignore all other distractors. After 20 s, the experimenter gives an acoustic signal, which tells the participants to move to the next line. The entire test duration is 4 min and 40 s. With no time constraints in the d2-R, virtually all subjects would solve all items correctly. However, the instruction to work as quickly and as accurately as possible leads to two types of errors: (1) omission errors, i.e., letters are omitted, which actually should have been crossed out, and (2) commission errors, i.e., letters have been struck through that should have been left out. Three main outcomes can be computed: the number of correct responses minus commission errors (i.e., focused attention), the total number of symbols processed (i.e., processing speed), and the percentage of all (commission and omission) errors made within the symbols processed (i.e., accuracy). Split-half reliability for the age-group of 11–12-year-olds (*r* = 0.80–0.91) and test-retest reliability with a time interval of 60–90 min (*r* = 0.75–0.92) assessed in the school setting has been shown to be acceptable (Brickenkamp et al., 2010).

Affect was assessed using the German short version of the PANAS-C (Ebesutani et al., 2012). The PANAS-C is a child-adapted version of the PANAS (Watson et al., 1988). The short version consists of 10 words that describe different feelings. Five items are related to positive affect (joyful, cheerful, happy, lively, proud) and five to negative affect (miserable, mad, afraid, scared, sad). Respondents have to indicate how they feel right now using a 5-point Likert scale ranging from 1 (“very slightly or not at all”) to 5 (“extremely”) on every item. The study by Ebesutani et al. (2012) yielded acceptable internal consistency estimates for the shortened positive affect (α = 0.89) and negative affect (α = 0.90) scales.

### Background variables

The German version (Watzlawik, 2009) of the Pubertal Development Scale (PDS; Petersen et al., 1988) was used to assess pubertal status. This scale consists of three questions for each gender, a sample question for boys being: “Have you noticed a deepening of your voice?” Response options are: not yet started (1 point); barely started (2 points); definitely started (3 points); seems complete (4 points). The response format varies by item. From the sum of the three items, the puberty index (ranging from 3 to 12) was calculated. Evidence for acceptable reliability and validity of the German version used in 9- to 13-year-old children has been provided by Watzlawik (2009).

Socioeconomic status was assessed using the Family Affluence Scale II (FAS II; Boudreau and Poulin, 2009). The scale consists of four questions asking children about things they are likely to know about their family (having their own bedroom at home, number of family-owned cars, computers, and number of family holidays in the past year). A sample item is: “Do you have your own bedroom for yourself?” Response options are: no (0 points); yes (1 point). The response format varies by item. The prosperity index (ranging from 0 to 9) was calculated from the sum of the four items. The scale has been demonstrated to have acceptable reliability and validity (Boudreau and Poulin, 2009).

The sport activity subscale (Block 5 + 6) of the Physical Activity, Exercise, and Sport Questionnaire (BSA; Fuchs et al., 2015) was used to assess children's physical activity level. In the sport activity subscale, respondents are asked to indicate up to three exercise types they regularly engage in and provide information about frequency (in the last 4 weeks) and average duration of each single activity. An activity level score in minutes per week was calculated based on the reports. Psychometric properties have been reported to be acceptable (Fuchs et al., 2015).

The BMI was calculated as the body weight (in kg) divided by the square of the height (in m).

### Statistical analyses

To test the successful experimental manipulation of physical exertion (PE) and cognitive engagement (CE), the four experimental conditions were grouped into low and high levels of PE and CE, resulting in two factors with two factor levels (2 × 2 design). In a next step, three separate analyses of variance (ANOVAs) were conducted with heart rate, RPE and RCE as dependent (manipulation check) variables. To test whether a potential change in children's attentional performance was due to the main effect of PE or CE or an interaction of the two (PE × CE), three separate ANCOVAs were conducted with the three attentional performance measures (focused attention, processing speed, accuracy) at post-test as dependent variables and the respective attentional performance pre-measures as covariates. In order to assess whether children's affective reaction to the treatments had an effect on their attentional performance, post-pre difference scores on the positive affect scale were included in the aforementioned ANCOVAs as additional covariates. The level of significance was set at *p* < 0.05 for all analyses. Partial eta square ($\eta_{\text{p}}^{2}$) was reported as an estimate of effect size.

To test the more explorative assumption that positive affect might be a potential mediator in the relationship between physical activity and attention, bias-corrected bootstrap analyses (95% BC confidence level; Bollen and Stine, 1992) were performed using AMOS Version 23, to reveal the indirect effects as significantly different from zero (Shrout and Bolger, 2002). The model-data fit was evaluated by comparing the calculated standard indices with the criteria for acceptable fit (Schermelleh-Engel et al., 2003): χ^2^ statistics; comparative fit index (CFI); the root mean square error of approximation (RMSEA); and the standardized root mean square residual (SRMR). To facilitate the comparison with other studies, all path coefficients are presented as standardized estimates.

## Results

### Manipulation check

The manipulation check revealed significant differences in heart rate, ratings of perceived physical exertion (RPE) and ratings of perceived cognitive engagement (RCE). Higher heart rates [*F*_(1, 90)_ = 359.94, *p* < 0.0005, $\eta_{\text{p}}^{2}$ = 0.800] as well as higher RPE [*F*_(1, 90)_ = 32.55, *p* < 0.0005, $\eta_{\text{p}}^{2}$ = 0.266] were observed in the two physically active compared to the sedentary conditions. The mean heart rate of the two physical activity groups corresponded to 73%, the mean heart rate of the two sedentary groups to 42% of the maximum heart rate (estimated using the formula 220–age; Fox et al., 1971). In the RCE, the conditions manipulated to be high in CE were perceived to be more cognitively engaging than the ones manipulated to be low in CE [*F*_(1, 90)_ = 14.62, *p* < 0.0005, $\eta_{\text{p}}^{2}$ = 0.140]. Taken together, the manipulation of PE and CE can be regarded as successful.

### Intervention effects

To test the first hypothesis, whether PE and CE impact children's attention either separately or in combination, three separate ANCOVAs were conducted with focused attention, processing speed, and accuracy, respectively, at post-test as dependent variables and the respective attentional performance pre-measures as covariates. ANCOVAs revealed that the high CE conditions elicited a better performance than the low CE conditions in focused attention [*F*_(4, 87)_ = 4.19, *p* = 0.044, $\eta_{\text{p}}^{2}$ = 0.046] but not in processing speed or in accuracy (*p*s > 0.05). Descriptive statistics of the dependent variables are presented in Table 2. No significant effects of the factor PE or the interaction of CE and PE were found in any of the applied measures of attention (*p*s > 0.05). These results indicate that a higher level of CE is responsible for small benefits (in terms of effect size) in focused attention. Non-significant results, especially for the accuracy measure as well as the reported effect sizes, will be discussed in more detail below.

**Table 2:** **Means (standard deviations in parenthesis) of the three attentional measures (focused attention, processing speed, accuracy), and positive affect of the four experimental conditions**.

	**High CE**		**Low CE**	
	**Combo group**	**Cognition group**	**Physical group**	**Control group**
**PRE-TEST**				
Focused attention	138.76 (23.25)	139.82 (18.43)	134.24 (14.78)	139.25 (18.76)
Processing speed	144.76 (23.96)	144.95 (18.55)	140.52 (14.23)	144.30 (18.37)
Accuracy	4.15 (2.64)	3.51 (4.06)	4.49 (3.27)	3.54 (2.80)
Positive affect	3.56 (0.93)	3.13 (0.95)	3.30 (0.64)	3.14 (0.90)
**POST-TEST**				
Focused attention	143.08 (22.97)	146.36 (17.08)	137.84 (18.75)	140.55 (18.01)
Processing speed	148.52 (23.62)	151.45 (17.31)	144.12 (19.08)	146.60 (17.80)
Accuracy	3.66 (2.45)	3.36 (2.97)	4.34 (3.56)	4.15 (2.86)
Positive affect	3.59 (0.90)	3.21 (1.02)	3.66 (1.01)	3.56 (0.87)

CE, cognitive engagement. In the d2-R test of attention; focused attention, number of correct responses minus commission errors; processing speed, total number of symbols processed; accuracy, percentage of all errors made within all symbols processed.

### Intervention effects with positive affect as a covariate

To test the second hypothesis, whether positive affect has an effect on children's attention, the change scores of positive affect (post - pre) were added as an additional covariate in the same models used in the main analyses. ANCOVAs revealed a significant main effect for the factor CE on focused attention [*F*_(5, 86)_ = 8.39, *p* = 0.005, $\eta_{\text{p}}^{2}$ = 0.089] as well as on processing speed [*F*_(5, 86)_ = 5.37, *p* = 0.023, $\eta_{\text{p}}^{2}$ = 0.059] but not on accuracy (*p* > 0.05). The change scores of positive affect had a significant effect with large effect size on focused attention [*F*_(5, 86)_ = 12.85, *p* = 0.001, $\eta_{\text{p}}^{2}$ = 0.130] and processing speed [*F*_(5, 86)_ = 12.67, *p* = 0.001, $\eta_{\text{p}}^{2}$ = 0.128], but not on accuracy (*p* > 0.05). No significant effects were found in any of the applied measures of attention for the factor PE or the interaction of PE and CE (*p*s > 0.05). These results indicate that changes in positive affect influence children's attention at post-test. When controlling for positive affect, the positive influence of high CE on both focused attention and processing speed is strengthened, as indicated by a large increase of the effect sizes.

### Mediation analyses

To test the third hypothesis, whether a potential relationship between the two manipulated variables (PE and CE) and attentional performance was mediated by changes in positive affect, mediation analyses were performed. Although mediation models for both PE and CE were run, only the CE models are presented here, as none of the PE mediation models were found to be statistically significant. Each model was set up with RCE as the independent variable, the post-test scores of focused attention, processing speed, and accuracy, respectively, as the dependent variables, and the gain scores of positive affect as the mediator. As in the intervention effect analyses, pre-test scores of the respective attentional measure was used as a covariate. Fit indices of the three models are presented in Table 3, all displaying a good model-data fit, with CFI, RMSEA, and SRMR satisfying the common critical values.

**Table 3:** **Goodness of fit statistics for the estimated models compared with recommendations for model evaluation by Schermelleh-Engel et al. (2003)**.

**Model**	**χ^2^**	***p* (df)**	**χ^2^/df**	**CFI**	**RMSEA**	**SRMR**
A.S.		≥0.05	≤3	≥0.95	≤0.08	≤0.10
Focused attention	0.21	0.650 (1)	0.21	1.00	<0.0005	0.019
Processing speed	0.01	0.911 (1)	0.01	1.00	<0.0005	0.005
Accuracy	0.86	0.354 (1)	0.86	1.00	<0.0005	0.035

A.S., Accepted Standard for Good Fit; CFI, Comparative Fit Index; RMSEA, Root Mean Square Error of Approximation; SRMR, Standardized Root Mean Square Residual.

**Figure 1:**
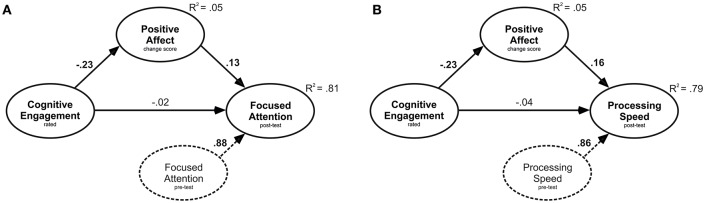
**Path diagram of the two models, with rated cognitive engagement as the predictor variable, positive affect change score as the mediator, and (A) focused attention and (B) processing speed, respectively, as the outcome variable**. All reported path coefficients (bold when significant, *p* < 0.05) are standardized estimates. Covariates are shown as dashed lines.

In the focused attention model (A), small but significant path coefficients are seen between the predictor RCE and positive affect, as well as between positive affect and focused attention (see Figure 1). Whereas the direct effect from RCE to focused attention was not significant, the indirect effect proved significant (β = −0.03, *p* = 0.020). The same pattern of results was found in the processing speed model (B): small but significant path coefficients between RCE and positive affect and between positive affect and processing speed. The direct effect from RCE to processing speed was not significant, but the indirect effect was (β = −0.04, *p* = 0.033). In the accuracy model, only the path coefficient between RCE and positive affect was significant (β = −0.23, *p* = 0.016). Positive affect was not related to accuracy (β = 0.03, *p* = 0.638). Neither the direct (β = 0.03, *p* = 0.723) nor the indirect effect (β = −0.007, *p* = 0.638) was significant. When the same procedures were performed using both pre-test and post-test scores of positive affect as a mediator, as well as using negative affect pre-test, post-test or change scores, none of the mediation analyses proved significant.

Taken together, the changes in positive affect induced by the experimental conditions mediated the effect between RCE and focused attention as well as between RCE and processing speed, but not the one between RCE and accuracy. This is in line with the effects found in the main analyses showing that only focused attention and processing speed are affected by the factor CE. Surprisingly, the relationship between RCE and positive affect was negative and not positive in nature. This issue will be discussed in more detail in Positive Affect as a Mediator.

## Discussion

The present study aimed to investigate the separate and/or combined effects of physical exertion (PE) and cognitive engagement (CE) induced by acute physical activity breaks on primary school children's attention. In sum, the results showed that (1) CE, but not PE, had a facilitating effect on children's focused attention and processing speed and that (2) changes in positive affect mediated the relationship between ratings of perceived cognitive engagement (RCE) and focused attention and processing speed, respectively. (3) The accuracy score remained unaffected. These results suggest that a short cognitively engaging activity contributes to children's attention at school. Specifically, involving primary school children in a challenging task for a period of 10 min leads to better performance, especially on those measures relying predominately on the speed component of the d2-R test of attention. From an applied perspective, the current findings could be interpreted as suggesting that one should not use physical activities, but rather cognitive engaging activities to immediately enhance children's attention at school. This interpretation would not be wrong, but only half the story. It is true that there was a main effect for CE, and none for PE. Nonetheless, it is important to note that a main effect in a 2 × 2 design always results from a combination of two experimental conditions. This means, the main effect of CE is also driven by the high-PE-high-CE condition. Therefore, until we know better, it is certainly not wrong to integrate physical activity breaks into the school day. On one hand, such activities seem to improve time-on task behavior (Howie et al., 2014) and no detrimental effects on attention have been published so far. On the other hand, they can lead to enhanced enjoyment (Vazou and Smiley-Oyen, 2014), an affective state associated with attentional performance. This positive association is in line with our second main finding. To the best of our knowledge, our study is the first to reveal positive affect as being a mediating variable in the relation between RCE and attention. This result underlines the importance of including measures for assessing the affective reactions in studies testing the effect of physical activity on children's attention.

### The main effect of cognitive engagement

The fact that CE was identified as the only factor leading to enhanced attentional performance in the current study, needs to be discussed first, since it contradicts our study predictions, which were based on the few recent studies investigating the role of CE inherent in physical activity. We expected the combination of PE and CE to have a stronger effect than PE or CE alone. However, independently of whether CE was induced by the sedentary completion of a trail-making test (high CE) or by running from one number painted on the floor to another (high CE, high PE), both resulted in enhanced attentional performance in the participating children. It is unlikely that increased arousal can explain this effect, since running around at different speeds (high PE) also led to increased arousal, yet no main effect was found for PE, nor an interaction between PE and CE. Given the similarity of the cognitive demands required by both the trail-making test and the d2-R test of attention (i.e., to discriminate simple visual stimuli, perform fast mental operations and react with an appropriate motor response), it can instead be assumed that the two conditions with high levels of CE pre-activated the same cognitive processes as used in the d2-R test of attention (Budde et al., 2008). Running, on the other hand, requires only the retrieval of automated motor control processes, without activating any of the aforementioned cognitive processes.

In contrast to our findings, acute intervention studies applying interventions of shorter duration in time and manipulating the amount of CE consistently found positive effects in favor of the cognitively engaging physical activity condition (Budde et al., 2008; Pesce et al., 2009; Benzing et al., in review). These studies compared a cognitively enriched condition with a less cognitively engaging physical activity (Budde et al., 2008; Pesce et al., 2009) or additionally with one passive control condition (Benzing et al., in review). This procedural approach is certainly appropriate for answering a research question of great practical relevance, namely whether cognitively enriched physical activity is equivalent or superior to simple aerobic exercise (containing only little CE). Practitioners are particularly concerned with the concrete indications as to how physical activity breaks should be addressed, with or without CE. However, if one wishes to answer the more theoretical questions, which of the two factors results in increased attentional performance, and whether there is a potential interaction between the two factors, a 2 × 2 design is essential. Best (2012), for example, systematically varied physical activity (physically active video games vs. sedentary video activities) and CE (challenging video games vs. repetitive video activities) in 6- to 10-years-olds. The study revealed a main effect of the physical component, but no main effect for CE nor any interaction. As an explanation for the absence of the main effect of CE and the interaction, he argued that the “marathon” game, used in the “low CE-high PE” condition, was more cognitively engaging than intended, as was supported by the children's activity engagement ratings. This is an important point, because being more cognitively challenging than planned, this condition facilitates finding a main effect of PE, but hinders finding a main effect of CE. In our study, we therefore tried to conceptualize the four conditions in an “additive manner” to systematically vary the amount of both CE and PE without cross-affecting the other dimension. Thus, whereas the “high CE-low PE” condition consisted of a sedentary trail-making test and the “low CE-high PE” condition of a simple running exercise, in the “high CE-high PE” condition these two tasks were combined in an exercise in which the children had to touch the numbers (placed on the ground) of the trail-making test by running through them. This procedure resulted in almost the exact same RCE scores in the two high-CE conditions, indicating that integrating a cognitive task into a physical activity is a viable means of systematically manipulating the CE component in studies investigating the relationship between physical activity and attention. Aside from the described differences in inducing CE, the shorter duration of our intervention (10 vs. 20 min) as well as the slightly lower heart rates in our physical conditions (149 vs. 156 bpm) may have been responsible for the diverging results between the current study and the study by Best (2012).

### The absence of the main effect of physical exertion

The absence of the main effect of PE might be explained by the duration of the intervention. The duration of 10 min was chosen because, due to time constraints and the pressure to cover the curriculum, teachers tend to implement only short physical activity breaks (Stylianou et al., 2016). As revealed by the meta-analysis by Chang et al. (2012), physical activity sessions lasting longer than 11 min are needed to positively affect cognitive functions immediately after physical activity, whereas shorter sessions had a negligible effect on cognitive performance. This meta-analytical finding is supported by the rare intervention studies comparing different durations of school-based physical activity. For example, comparing a physical activity break of 5 min and a physical education class lasting 30 min with a control group listening to an audiobook, Kubesch et al. (2009) revealed positive effects on inhibitory attention for the physical education class, but not for the physical activity break. They discussed that the 5-min physical activity break intervention was not long enough to increase attentional performance (measured using a flanker task). Concerning classroom-based physical activity, the dose-response relation has to date only been investigated by the Brain BITES study, in which physical activity breaks of 5, 10, and 20 min duration were compared with a sedentary condition (Howie et al., 2014, 2015). Whereas positive effects on on-task behavior were found after the 10 min, but not after the 5 and 20 min intervention (Howie et al., 2014), none of these interventions of different durations were effective in enhancing children's attention, measured using the Trail Making Test (Howie et al., 2015). Thus, it seems that physical activity breaks of 10 min duration, which “require little thought” (Diamond, 2015) are not suitable for enhancing children's attentional performance. This conclusion is in part supported by a recent study, not in children but in healthy young men, in which the dose–response relation between exercise duration and attention was tested (Chang et al., 2015). The authors reported that exercising for 20 min at moderate intensity on a cycle ergometer resulted in better attentional performance than exercising for 10 or 45 min. This result suggests a curvilinear relationship between exercise duration and attention. However, since physical activity breaks of 20 min duration are hard to implement at school, the addition of a cognitive component in a shorter physical activity break could indeed be a promising means of enhancing children's attention.

The aforementioned curvilinear relationship between exercise duration and attention may be also important in the relation between the duration of a cognitively engaging activity and the following attentional performance. Studies which have compared a cognitively engaging intervention with a less engaging one to examine the impact on children's attention have used physical activity durations of 10 min (Budde et al., 2008), 15 min (Benzing et al., in review) 20 min (Jäger et al., 2015), 45 min (Schmidt et al., 2015a) or 50 min (Gallotta et al., 2012, 2015). Whereas shorter durations of cognitively engaging physical activities proved to have a facilitating effect on attentional performance (Budde et al., 2008; Benzing et al., in review), no intervention lasting longer than 15 min produced a positive effect in favor of the cognitively engaging condition. Explaining the facilitating effects of cognitively engaging physical activity by means of a pre-activation of shared cognitive processes, the detrimental effects after longer lasting interventions (Gallotta et al., 2012, 2015) may be explained with the help of the strength model of self-control (Baumeister et al., 1998). Since cognitively engaging physical activity is conceptualized as an activity that uses control processes (Pesce, 2012) and control processes, in turn, are costly in terms of consuming inner resources, the shared common capacity-limited reservoir of voluntary attention or mental effort (Audiffren and André, 2015) may be depleted after prolonged physical activity with CE. Using the analogy of self-control as a muscle which gets tired through exhaustion, but recovers after a delay (Muraven and Baumeister, 2000), one might speculate about the existence of an inverted u-shaped function between CE and attentional performance as well. Referring back to the muscle analogy, no athlete would take part in a competition without warming-up nor putting a great strain on specifically those muscles used in the subsequent contest. Thus, maybe as in sports, there is an optimal “warm-up” duration for CE to pre-activate the same processes before using them in subsequent cognitive tasks. These speculations could be tested by applying the same design as used, for example, to examine the dose-response relation between exercise duration and attention (Chang et al., 2015). However, in doing so one should individualize the CE of the activity to the same extent as is possible for PE. Only such an individualized approach would enable researchers to properly investigate the dose-response relation between cognitively engaging physical activity and attention.

### No effects on accuracy

The absence of an effect on the test's accuracy component is in line with the results of Gallotta et al. (2012, 2015), investigating 8–11-year-olds as well as with the findings of Schmidt et al. (2015a), testing 10–12-year-olds. However, it contradicts the findings of Budde et al. (2008), who found large effects on 13–16-year-old adolescents' attentional performance in all three attentional measures. One explanation for these disparate findings could be the different participants' age ranges in the aforementioned studies. Developmental data suggests that with increasing age, older children start slowing down their responses in challenging cognitive tasks to prevent a deterioration in accuracy (Davidson et al., 2006). This emerging speed-accuracy trade-off is explained by improved metacognition through development (Best, 2010). Thus, it is likely that, in younger children, the accuracy score of the d2-R test of attention is less sensitive to the effects of acute physical activity than in adolescents. Nonetheless, in the present study the positive effects on focused attention and processing speed was at least not detrimental to the accuracy, as would be expected based on a meta-analysis investigating the effects of acute exercise on the speed and accuracy component in cognitive tasks (McMorris et al., 2011).

### Positive affect as a mediator

Revealing positive affect as a mediator supports the postulated importance of affective reactions in the relationship between physical activity and cognition (Audiffren and André, 2015; Diamond and Ling, 2016). However, to be honest, we were astonished (a) to find positive affect gain scores as a mediator between RCE and focused attention but not between PE and focused attention and (b) to find a negative correlation between RCE and positive affect changes, but a positive correlation between affect and attention. As far as we know, our study is the first to provide empirical evidence for the mediating role of positive affect in the relation between physical activity and cognition, which has to be discussed in the light of theories predicting facilitating or deteriorative effects of positive affect on attention.

The fact that positive affect (change score) was positively—and not negatively—related to attention can best be explained by *mood as a facilitator theories* (Isen, 1999, 2008; Forgas and Eich, 2012), since they predict enhanced performance in interesting and novel tasks when one is in a positive affected mood. Neither *capacity limitation theories* (e.g., Seibert and Ellis, 1991), nor *mood as information theories* (e.g., Schwarz, 1990) would predict a facilitative effect of positive affect gain scores on focused attention. The former assume that any induced affective mood state would impair attention compared to neutral affective states, since less cognitive resources would be available for a cognitive task. The latter suppose that positive affect generally leads to heuristic processing, characterized by fast but imprecise information processing, which would worsen performance especially in attentional tasks. However, it should be pointed out that other factors apart from the valence dimension of affect could have influenced our results. Motivational intensity, that is, whether the affect is associated with a motivation to approach or avoid a certain stimulus, has been shown to have an impact on cognitive functioning (Gable and Harmon-Jones, 2010). Whereas some positive affective states are relatively low in approach motivation (e.g., joy after listening to a pleasant story), others are relatively high in approach motivation (e.g., enthusiasm while trying to improve an individual test score). Affect with a low motivational intensity broadens and affect with a high motivational intensity narrows the attentional focus (Harmon-Jones et al., 2013). Taking into account that the interventions of the combo group and the cognition group were much more goal-oriented and challenging—remember, that they were asked to link the numbers as quickly as possible and to improve during the second period—compared to the interventions of the physical group and the control group, it may be speculated that the former led to affective states with a higher motivational intensity than the latter. Motivational changes induced by specific tasks should therefore be considered or even manipulated systematically in future studies searching for qualitative characteristics (not only) of specific physical activities that potentially affect attention.

The results regarding the relationship between RCE and positive affect are difficult to interpret since we found a negative correlation between the two variables. Thus, it seems that an increase in CE goes hand in hand with a reduction of positive affect. At first sight, this result seems somehow contradictory considering that (1) CE was shown to be the factor leading to increased attentional performance and (2) positive affect was shown to promote attentional performance. Referring to the Yerkes-Dodson law, which originally described the relationship between arousal and performance (Yerkes and Dodson, 1908), one way to resolve this contradiction is the assumption of an optimal cognitive load rather than a maximal cognitive load in order to promote positive affect and attention. A differential perspective is needed to test this inverted u-shape hypothesis because the cognitively optimal challenge point might depend on each individual's cognitive capacity. In fact, the matching of task difficulty (e.g., challenge) and individual abilities could be of major relevance, because a better match is expected to produce greater enjoyment (Abuhamdeh and Csikszentmihalyi, 2012), which is a psychological state leading to positive affect (Kimiecik and Harris, 1996). Furthermore, children can be assumed to differ in their *need for cognition*, which is defined as an “individual's tendency to engage in and enjoy effortful cognitive processing” (Cacioppo et al., 1984, p. 306). Due to the small sample size, we were unable to conduct differential analyses. This perspective could however be investigated in future studies.

### Limitations and future directions

Like any study, the current one also has certain limitations, which have to be addressed. First, when performing the manipulation check no standardized instrument was used to control for induced CE. The usability of the rating of perceived CE has been proven to be feasible to distinguish between two physical activities with diverging degrees of cognitive demands in adolescents (Benzing et al., in review). However, one might critically ask whether children at the age of 11–12 years are able to correctly estimate CE inherent in physical activity. Developmental studies on children's metacognitive abilities would at least suggest that this is the case, since they show that children from the age of 8 years do quite well in tasks requiring introspection, such as being aware of their own thoughts (Flavell et al., 2000) or monitoring their own academic performance (Roebers et al., 2009). Considering that the verbal anchors were chosen to be identical to those of the Borg RPE scale, which has been shown to have acceptable reliability and validity in preadolescents, it is probable that the adapted RPE scale is a viable tool for ecologically assessing CE in child populations too.

Second, neither the level of cognitive demand, nor PE was adjusted on an individual level in the current study. The cognitively engaging physical activity of the current study can be regarded as rather high in cognitive demands as supported by subjective ratings. Therefore, depending on their cognitive abilities, some subjects might have been cognitively under- or overtaxed resulting in a lowered attentional performance after the high-CE conditions. In earlier studies, the lack of effect in children with lower academic achievement has already been discussed in terms of a possible depletion of self-control resources (Schmeichel, 2007) due to an attentional overload for exactly those children (Jäger et al., 2015). Following this line of argument, and extending it to the physical dimension, we cannot be sure that all individuals were challenged with an optimal level of both physical and cognitive exertion. This might have resulted in an underestimation of the potential benefits for children's attention.

Third, in the current study, a between-subjects instead of a within-subjects design (Best, 2012) was used. Even though our analyses revealed no significant differences in key variables between the four groups, subjects inevitably differ in many other, unmeasured personal characteristics. Whereas in between-subjects designs, these differences are treated as error, in within-subjects designs, the subjects serve as their own controls and differences among subjects can be separated from the error. The latter design obviously leads to greater statistical power to detect potential intervention effects. However, in the present study the children would have had to complete the d2-R test of attention five times, which could have led to participants' dropping out before completion of the study.

An important direction for future research examining the specific contribution of CE inherent in physical activity to children's attention involves the development of a validated instrument for measuring CE. Recent studies have attempted to measure CE using either newly developed observational data (Schmidt et al., 2015b), psychophysiological measures or subjective ratings of perceived CE (Benzing et al., in review). Despite these efforts, research still lacks a reliable and sensitive means of assessing the level of CE (Tomporowski et al., 2015). Such an instrument would, for example, enable researchers to adjust the cognitive load of a task to the performance level of the child participating in the study. This is important since it has been shown that matching the optimal challenge point is crucial for promoting cognitive development in childhood (Pesce et al., 2013). To develop a child-appropriate instrument, further research could utilize the items from the NASA Task Load Index (Hart and Staveland, 1988), a multidimensional assessment tool for rating perceived workload, and combine these with the Self-Assessment Manikin (Bradley and Lang, 1994), a non-verbal pictorial assessment technique usually used to measure a person's affective reactions. However, both the applicability in field research and the usability in child samples will need to be evaluated.

Besides CE, there are at least two other dimensions of the general engagement construct (Fredricks et al., 2004) which could influence consequent attentional performance: behavioral engagement (i.e., persistence and concentration) and emotional engagement (i.e., affective reactions such as enjoyment or pleasure), both linked to attentional control (Diamond and Ling, 2016). For example, a recent study has shown that a classroom-based physical activity intervention increased children's attention and enjoyment, the latter being a part of *emotional* engagement (Vazou and Smiley-Oyen, 2014). To the best of our knowledge, no study has ever tested whether classroom-based physical activities could potentially also lead to increased *behavioral* engagement. Since academic lessons accompanied by physical activities in the classroom are perceived by primary school children as being more enjoyable than traditional academic lessons (Vazou et al., 2012), one might speculate that children would persist more in their academic activities. Therefore, researchers might be well advised to aim not only to improve attentional performance directly, but also to consider those variables that influence attentional control indirectly. Further studies could broaden the narrow focus on CE to include emotional and behavioral engagement, as well as more of the qualitative characteristics of physical activity, to reveal how they are related to cognitive performance. This would hopefully lead to interventions, which are both effective in fostering cognitive development and enjoyable for the teachers and children taking part in them.

## Author contributions

MS designed the study, analyzed the data and wrote the manuscript. VB did the manipulation check and intervention effect analyses and helped writing the manuscript. MK contributed to designing the study and writing the manuscript, did and managed data collection. All authors agree to be accountable for the content of the work.

### Conflict of interest statement

The authors declare that the research was conducted in the absence of any commercial or financial relationships that could be construed as a potential conflict of interest. The reviewer KG and the handling Editor declared their shared affiliation, and the handling Editor states that the process nevertheless met the standards of a fair and objective review.
